# Microbial architecture and metabolic profiles in *Xiaoqu*: insights into traditional glutinous rice wine fermentation

**DOI:** 10.3389/fmicb.2026.1869908

**Published:** 2026-06-10

**Authors:** Baoyu Peng, Jinghan Shu, Bifeng Chen, Changchun Li, Yu Han

**Affiliations:** 1 Hubei Urban-Rural Social Work and Social Governance Modernization Research Center, Hubei Engineering University, Xiaogan, China; 2 Hubei Key Laboratory of Resource Utilization and Quality Control of Characteristic Crops, College of Life Science and Technology, Hubei Engineering University, Xiaogan, China; 3 College of Life Science and Technology, Hubei Engineering University, Xiaogan, China; 4 Research Center of Hubei Small Town Development, Hubei Engineering University, Xiaogan, China; 5 School of Architecture, Hubei Engineering University, Xiaogan, China

**Keywords:** community assembly, glutinous rice wine, metabolic modulation, microbial architecture, solid-state fermentation, *Xiaoqu*

## Abstract

*Xiaoqu* is a traditionally propagated multi-species starter used in Chinese sweet glutinous rice wine fermentation and represents a low-temperature solid-state fermentation system distinct from Japanese pure-culture *Aspergillus oryzae koji* and Western malt-based saccharification platforms. Unlike defined inoculation systems, *Xiaoqu* functions as a process-shaped microbial architecture assembled through repeated ecological selection under artisanal production conditions. This review synthesizes current knowledge regarding the microbial ecology, guild organization, fermentation trajectories, and metabolite modulation associated with *Xiaoqu*-mediated glutinous rice wine production. *Xiaoqu* communities are typically structured around amylolytic molds, fermentative yeasts, non-*Saccharomyces* yeasts, and lactic acid bacteria (LAB), which collectively regulate saccharification, acidification, ethanol restraint, and flavor formation through coupled ecological and metabolic interactions. We propose a process-phytochemical selection framework, presented as a literature-derived conceptual hypothesis, to explain how botanical inputs, matrix properties, and processing conditions jointly shape microbial succession, interaction topology, and metabolic trajectories. The review further develops an architecture-to-trajectory perspective in which sugar-acid-ethanol coupling emerges as a systems-level phenotype governed by guild interactions, cross-feeding, feedback regulation, and environmental constraints rather than by individual taxa alone. Functional redundancy, hybrid starter systems, temporal trajectory analysis, and safety constraints associated with toxigenic risks are also discussed as key factors influencing fermentation robustness, reproducibility, and sensory stability. Finally, we highlight the need for architecture-preserving control strategies integrating strain-level multi-omics, synthetic-community reconstruction, and trajectory-oriented process analysis to support mechanistic understanding and controllable design of *Xiaoqu*-mediated fermentations.

## Graphical abstract

**Figure:**
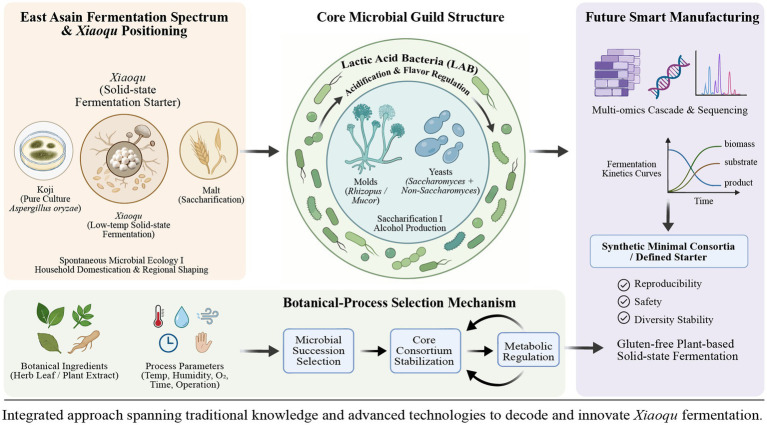

## Highlights

- *Xiaoqu* is a traditionally propagated multi-species starter distinct from Japanese *koji* and Western malt-based systems.
- Molds, yeasts, and lactic acid bacteria jointly regulate saccharification, acidification, ethanol restraint, and flavor formation.
- Sugar–acid–ethanol coupling emerges from guild interactions, cross-feeding, and metabolic rerouting within process-shaped microbial architectures.
- Multi-omics and architecture-preserving control support reproducible, safe, and functionally robust *Xiaoqu*-based fermentations.

## 1 Introduction

Glutinous rice wine fermentation is a core starch-based bioprocess in East Asia, yet it is conceptually incompatible with the mainstream fermentation paradigms for defined-input systems (Peng et al., 2025). In Western brewing and distilling, saccharification depends on malt-derived enzymatic activity and fermentation on standardized yeast inoculants, allowing process performance to be interpreted via controllable kinetics and relatively fixed inputs (Moyano et al., 2024; Xiong Q. et al., 2025; Almeida et al., 2026). Japanese *Koji*-based fermentation systems also follow the defined-input logic, where *Aspergillus oryzae* propagation generates a controllable saccharifying module for combination with selected yeasts (Lee da et al., 2016). By contrast, *Xiaoqu*-mediated fermentation adheres to a distinct mechanism: a low-temperature solid-state starter is prepared and applied under ambient conditions, and its “starter identity” is reflected not in a stable strain profile but in the recurrent community assembly outcome under weak external control (Yan et al., 2024). This difference transcends taxonomic and craft-level distinctions. Most glutinous rice wine styles require specific fermentation phenotypes—rapid saccharification with sweetness retention, moderate acidification and controlled ethanol accumulation—and these traits are stably achieved despite fluctuations in inoculum composition and fermentation environment (Mao et al., 2023). However, existing scientific research on *Xiaoqu* remains largely descriptive, focusing on raw materials, regional characteristics and static microbial inventories, with limited ability to explain the maintenance of stable functional performance across different contexts. Thus, it is imperative to construct a mechanistic framework that regards *Xiaoqu* as a functional system orchestrating microbial activities to yield predictable fermentation outcomes.

Such a framework begins with microbial architecture. Here, microbial architecture denotes the community-level organization of *Xiaoqu* microbiota and is operationally defined by three dimensions: (i) the composition and relative abundance of functional guilds, (ii) the topology of interactions among guilds, and (iii) the spatial and physicochemical constraints imposed by the structured rice-derived matrix (Zhang et al., 2024). Functional guilds refer to groups of microorganisms that perform similar ecological functions irrespective of taxonomic identity and may be characterized by indicators such as functional roles, metabolic outputs, or substrate utilization patterns. In *Xiaoqu*, representative guilds include amylolytic molds, fermentative yeasts, and acid-producing bacteria. Guild structure therefore describes the organization of these functional groups in terms of their relative abundance, diversity, redundancy, and succession dynamics. Interaction topology refers to the organization of ecological relationships among guilds and may be represented using interaction-network properties such as connectivity, modularity, centrality, and interaction strength.

In *Xiaoqu*, molds, yeasts, and bacteria do not simply coexist; they co-construct the biochemical environment that governs fermentation trajectories (Gong et al., 2024). Amylolytic molds release sugars from starch and can remodel microhabitats through hyphal growth, shaping diffusion and niche formation. Yeasts convert sugars into ethanol and diverse secondary metabolites, but their metabolic flux is constrained by sugar timing, pH, oxygen availability, and inhibitory environments established by other guilds. Bacteria—often including lactic acid bacteria (LAB)—modulate pH and succession, influencing both microbial stability and sensory balance. These guilds are coupled through cross-feeding, pH-mediated regulation, and competition for oxygen and limiting nutrients (Jiang et al., 2026). This perspective shifts the unit of explanation from “key species” to organized consortia and provides a parsimonious resolution to a central paradox: *Xiaoqu* communities can vary taxonomically across producers and regions while maintaining stable process-level performance, implying that conserved function is encoded in guild structure, redundancy, and interaction constraints rather than strict taxonomic identity. In *Xiaoqu*, organization—not membership—most plausibly predicts function.

Microbial architecture matters because it generates metabolic modulation, the second concept required to explain glutinous rice wine phenotypes. Fermentation in these systems is not a single conversion of starch to ethanol; it is a dynamic chemical trajectory in which sugars, acids, and ethanol both reflect and regulate microbial activity (Peng et al., 2026b). In *Xiaoqu*-mediated fermentations, saccharification is often rapid, producing time-varying sugar landscapes rather than a single end-point concentration. Acidification is not merely a by-product; it functions as a control layer that regulates enzyme activity, suppresses undesirable growth, and tunes sensory balance (Peng et al., 2026b). Notably, ethanol accumulation can remain restrained despite efficient saccharification, suggesting that alcohol yield is frequently an emergent property of consortium constraints and feedbacks rather than a direct readout of sugar availability. Metabolic modulation therefore emphasizes trajectories—how sugar release, pH kinetics, and ethanol production are coordinated over time through community interactions—and it highlights why end-point measurements alone are insufficient: similar final chemistry can arise from different pathways with distinct implications for stability and sensory outcomes (Ruan et al., 2026). Understanding *Xiaoqu* thus requires connecting architecture to trajectories, rather than linking taxa to isolated endpoints.

This architecture-to-trajectory logic implies that *Xiaoqu* is best viewed as a process-shaped ecosystem. Unlike starters produced by defined inoculation under standardized conditions, *Xiaoqu* is assembled through recurring steps—mixing, shaping, incubation, drying, and storage—that impose ecological filters (Xia et al., 2023). These steps generate structured microenvironments with gradients in temperature, moisture, oxygen, and metabolites, selecting physiological traits consistent with low-temperature function and robustness to variability. The matrix is not inert; it governs diffusion and niche partitioning and enables (or restricts) hyphal scaffolding that can stabilize guild coexistence. In some traditions, botanicals add a further layer of selection. Rather than treating botanicals as generic antimicrobials, it is more informative to view them within a process–phytochemical selection landscape, where plant chemistry interacts with temperature–moisture–oxygen regimes to steer succession, suppress opportunists, and stabilize functional cores (Zheng et al., 2025). Geography and seasonality act as boundary conditions that diversify community membership by altering ambient microbial pools and assembly constraints, yet fermentation phenotypes can converge, indicating that robustness is supported by functional redundancy and architectural constraints that channel community dynamics toward productive states (Lu et al., 2017; Yu et al., 2021a; Yu et al., 2023a; Lei et al., 2024). Here, functional redundancy does not imply complete taxonomic interchangeability, but rather partial overlap in functional capacity among taxa within the same guild. In *Xiaoqu*, redundancy may arise through three mechanisms: overlap in enzymatic functions (e.g., starch hydrolysis, acid production, or precursor transformation), niche overlap among taxa occupying similar physicochemical environments, and alternative metabolic pathways that generate comparable fermentation outputs (Zong et al., 2026). Such redundancy is consistent with ecological insurance theory, whereby overlapping functions buffer process performance against compositional turnover. However, redundancy is not cost-free; functionally similar taxa may compete for limiting substrates and oxygen, while shifts in dominant members may reroute metabolic fluxes and alter secondary metabolite profiles (Jiang et al., 2026). Therefore, functional stability emerges not from fixed membership, but from redundancy operating under architectural constraints. In this view, processing and environment constitute the hidden “inoculation”: they do not merely host microbes; they actively assemble the architecture that enables metabolic modulation.

**Figure 1:**
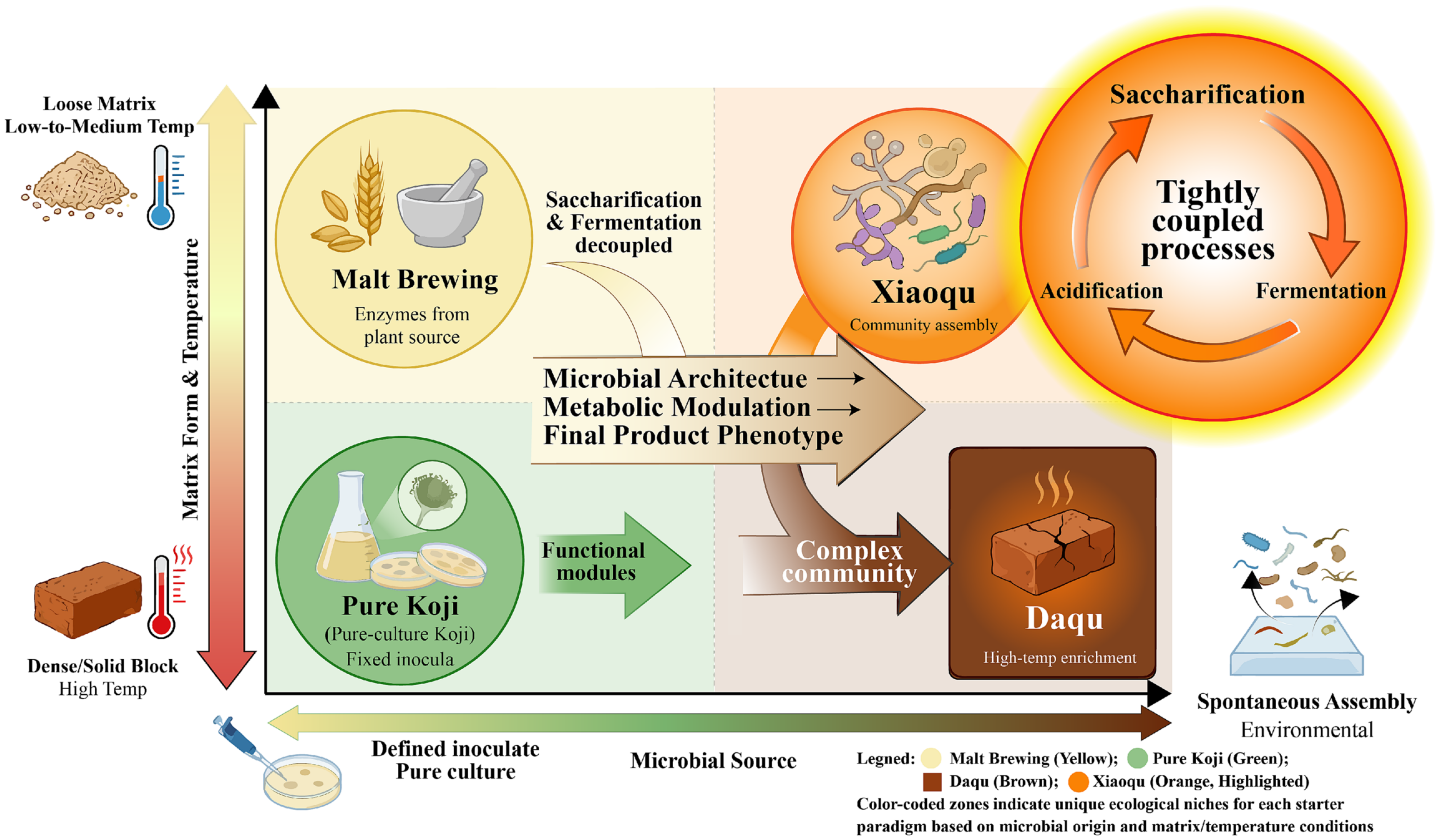
Major starter paradigms for rice wine fermentation and their contrasting assembly logics. The paradigms are presented as idealized assembly logics rather than rigid categories; hybrid starters may occupy intermediate positions.

A useful way to clarify why *Xiaoqu* resists conventional starter definitions is to contrast it with dominant “defined-input” paradigms in starch fermentation. In malt-based brewing and distilling, saccharification is primarily delivered by malt-derived enzymes and alcoholic fermentation by standardized yeast inoculate, enabling performance to be interpreted from fixed inputs and controllable kinetics (Yang et al., 2021). Pure-culture *Koji* platforms follow a similar modular logic, where propagation of *Aspergillus oryzae* provides a relatively controllable saccharification module that can be paired with selected yeasts (Peng et al., 2024). *Xiaoqu*, in contrast, is best understood as a process-shaped starter whose identity emerges from repeated community assembly within a structured rice matrix under low-to-moderate temperature regimes. This shift in logic—defined inputs versus process-shaped assembly—repositions *Xiaoqu* from a “starter composition” problem to a microbial architecture problem, where guild organization and interaction constraints govern metabolic modulation along sugar–acid–ethanol trajectories (see Figure 1). Importantly, these paradigms should be viewed as conceptual endpoints rather than mutually exclusive categories. Intermediate or hybrid systems, including enzyme-supplemented *Qu*, mixed *Qu*, and defined multi-strain starters, may integrate elements of modular control and open community assembly, thereby occupying transitional positions along the starter-design continuum (Yang et al., 2023; Xia et al., 2023; Xiong Z. et al., 2025; Peng et al., 2026a). In addition, *Daqu* represents another complex-community paradigm characterized by high-temperature assembly and dense matrix structure, extending the comparison beyond low-temperature *Xiaoqu* systems and providing an additional ecological reference point (Chen Z. et al., 2026) (see Figure 1).

Despite rapid growth in sequencing and metabolite profiling, *Xiaoqu* research still faces a familiar bottleneck: rich correlations but weak causality. Community surveys and end-point chemistry have established broad guild membership and candidate associations with sensory or fermentation outcomes, but fewer studies resolve how spatiotemporal organization within starters translates into fermentation kinetics and mechanistic trajectories (Liu et al., 2022; Feng et al., 2024; Chen L. et al., 2026). Advancing the field requires integrative designs that anchor multi-omics to time-resolved sugar–acid–ethanol dynamics, quantify guild structure and redundancy rather than rely on taxa lists alone, and measure microenvironmental gradients that stabilize or destabilize interaction networks (Zeng et al., 2025; Ruan et al., 2026). Experimentally tractable reconstruction of minimal yet functional consortia—guided by guild organization and interaction constraints—will be essential to identify which architectural features are necessary and sufficient for desired metabolic modulation. Against this backdrop, this review synthesizes current evidence through an architecture-to-trajectory lens, clarifying the scope of *Xiaoqu*, the eco-evolutionary and process drivers of its assembly, and the interaction mechanisms that translate consortium organization into glutinous rice wine fermentation performance. A mechanistic *Xiaoqu* science will therefore emerge not from deeper inventories alone, but from coupling ecology, kinetics, and reconstruction to explain—and ultimately control—community-driven metabolic modulation.

## 2 Scope and definition of *Xiaoqu*

### 2.1 Positioning *Xiaoqu*

Rice wine fermentations in East Asia rely on starter systems that integrate saccharification and fermentation in ways that differ fundamentally from malt-based brewing and pure-culture *Koji* platforms. In the Chinese context, starters are often grouped under *Jiuqu*; however, this umbrella term covers systems that diverge in their assembly environments, microbial ecologies, and functional priorities. *Xiaoqu* occupies a distinctive niche within this landscape and therefore warrants focused synthesis. It is typically rice-based, produced under low-to-moderate temperature regimes, and frequently assembled through traditional or semi-controlled inoculation (Yan et al., 2024). These features create an ecological regime that contrasts with high-temperature, large-format starters tailored for thermophilic solid-state processes, where heat selection and prolonged incubation tend to favor different physiological strategies and community dynamics (Ali et al., 2024; Xu Y. et al., 2025). Consequently, *Xiaoqu* cannot be treated as a scaled-down analog of other starter types. Rather, it is a starter whose mild assembly conditions and rice-matrix habitat select for a characteristic balance among saccharification, acidification, and fermentation—precisely the balance that underpins the fermentation phenotype sought in many glutinous rice wine styles (Gao et al., 2024; Zheng et al., 2025).

Within the broad *Jiuqu* landscape, *Xiaoqu* occupies a distinct ecological niche because its assembly conditions and matrix structure bias community organization toward low-temperature saccharification–fermentation coupling rather than thermophilic succession (Song et al., 2025). Positioning *Xiaoqu* alongside malt- and *Koji*-based paradigms underscores that the critical distinction is not cultural origin but assembly logic: *Xiaoqu* expresses functionality through ecologically assembled consortia shaped by process filters and microenvironmental gradients, whereas defined-input systems externalize control through standardized enzymes and inocula (Figure 1). This conceptual positioning motivates an architecture-to-trajectory framework for explaining how similar glutinous rice wine phenotypes can emerge despite taxonomic variability.

Positioning *Xiaoqu* in a mechanistic review also requires defining scope with enough precision to avoid conceptual drift. Here, *Xiaoqu* refers to rice-matrix starters assembled under relatively mild thermal conditions and widely used for sweet rice fermentations and glutinous rice wine systems (Chen et al., 2021a; Zhang W. D. et al., 2022; Wan et al., 2025; Zhang et al., 2025c). This boundary is intentionally narrow. A broad survey of *Jiuqu* risks conflating assembly regimes and importing assumptions from thermophilic or otherwise distinct processes, which can obscure the logic of microbial organization and dilute trajectory-level interpretations. A *Xiaoqu*-centered scope, in contrast, enables testable hypotheses about how low-temperature assembly, structured microenvironments, and repeated practice stabilize a microbial architecture capable of producing rapid saccharification with sweetness retention, controlled acidification, and restrained ethanol accumulation. In other words, a narrow scope is not a limitation but a prerequisite for explaining how *Xiaoqu* links microbial architecture to metabolic modulation in glutinous rice wine fermentation.

### 2.2 From composition to consortium

A recurring limitation in the literature is the tendency to define *Xiaoqu* by composition—either by ingredient lists (rice flour, water, optional botanicals) or by taxa inventories. Such descriptions are valuable as context, but they rarely predict fermentation performance. Ingredient lists fail because identical materials can yield different starters when processing filters and ambient environments differ; taxa lists fail because similar fermentation outcomes can be delivered by different community memberships (Chen et al., 2021a; Zhao et al., 2022; Chen et al., 2023a; Chen et al., 2023b; Song et al., 2025). *Xiaoqu*, assembled through traditional or semi-controlled inoculation, makes this limitation unavoidable: taxonomic membership is inherently variable across time and place, yet desirable fermentation phenotypes can remain conserved. This observation motivates a shift from composition to consortium thinking, where the explanatory focus moves from “what is present” to “how functions are organized and coupled.”

Accordingly, *Xiaoqu* is defined here as a naturally assembled microbial architecture: a structured consortium organized into interacting functional guilds, embedded in a rice-derived matrix, and stabilized by process and environmental constraints (Sakandar et al., 2020; Kang et al., 2022; Shen et al., 2025). Three guilds are repeatedly implicated and are best interpreted as coupled modules rather than independent contributors. Amylolytic molds provide saccharifying enzymes and, through hyphal growth, can restructure microhabitats by altering porosity, diffusion pathways, and oxygen micro-gradients. Fermentative yeasts convert sugars into ethanol and diverse secondary metabolites, but their metabolic flux is shaped by the timing and localization of sugar release, by pH, and by inhibitory environments generated within the consortium. Bacteria—frequently including LAB—modulate pH and succession and can influence both microbial stability and sensory balance. The defining feature is not merely the coexistence of these guilds, but their coupling: mold-driven sugar release constructs the substrate landscape; bacterial acidification regulates enzyme activity and competitive hierarchies; and yeast metabolism generates metabolites and redox shifts that feed back into bacterial dynamics and, indirectly, mold performance. Functional performance therefore emerges at the consortium level, where interaction topology governs trajectory-level outcomes.

This architectural framing also clarifies what “core microbiome” should mean in *Xiaoqu* (Chen et al., 2021a; Miao et al., 2022; Tian et al., 2022; Liu et al., 2023; Zhu et al., 2023). A strict taxonomic core is often elusive, particularly across regions and producers, and may not even be the most meaningful target. A functional core is more plausible: the repeated representation of saccharification, fermentation, and stabilization functions, even when carried by different taxa (Xia et al., 2023). Functional redundancy within guilds—including overlap in enzymatic functions, ecological niches, and alternative metabolic pathways—can buffer membership variation, while interaction constraints and microenvironmental gradients can channel succession toward productive states. This is the basis for understanding why *Xiaoqu* can be taxonomically diverse yet functionally stable, and why linking community profiles to end-point chemistry alone often yields weak mechanistic insight. Treating *Xiaoqu* identity as residing in guild structure, interaction topology, and process-shaped assembly filters establishes a coherent pathway for explaining how microbial architecture gives rise to metabolic modulation—and why this linkage is central to glutinous rice wine fermentation phenotypes. These distinctions highlight that *Xiaoqu* occupies a starter paradigm that is ecologically and functionally distinct from both thermophilic solid-state starters and defined-input platforms, warranting a dedicated framework for comparison (Table 1).

**Table 1:** Comparative positioning of *Xiaoqu* within major starter paradigms relevant to rice wine fermentation.

Starter paradigm	Assembly regime (typical)	Matrix and microenvironment	Architectural organization (guild logic)	Dominant functional emphasis	Expected phenotype on rice substrates (trajectory-level)	Controllability and typical risks	References
*Xiaoqu* (rice-based, low-temperature)	Ambient/low–moderate temperature; short–moderate incubation; drying/storage stabilize	Rice-derived solid matrix; strong oxygen/moisture gradients; potential hyphal scaffolding; optional botanicals as selection modifiers	Multi-guild consortium: amylolytic molds and fermentative yeasts and acidifying/stabilizing bacteria; redundancy common	Coupled saccharification–fermentation with ecological stabilization	Rapid saccharification; controlled acidification; ethanol often restrained/plateaued in sweet-style trajectories; sensory complexity	Medium controllability; batch variability; safety depends on hygiene and pH/trajectory control; pipeline comparability limits interpretation	Lv et al. (2012), Shin et al. (2017), Wang et al. (2019), Peng et al. (2023), Wang et al. (2023), Gao et al. (2024)
*Daqu* (large-format, higher temperature solid-state starters)	Higher temperature, longer maturation; strong thermal selection	Thick bricks; pronounced thermal gradients; prolonged aging selects stress-tolerant taxa	Complex communities; often thermotolerant/thermophilic guilds; architecture shaped by heat and long succession	Strong flavor development plus fermentation support in distilled spirits contexts	Not optimized for sweet rice wine; may yield stronger alcohol/acid trajectories; different aroma logic	Lower-to-medium controllability; long process; contamination risk managed by heat selection but still variable	Zhang J. et al. (2022), Yang and Zhou (2025), Ren et al. (2025)
Herbal *Xiaoqu* variants	Similar to *Xiaoqu*; additional botanical inputs	Same as *Xiaoqu* plus phytochemical constraints	Multi-guild consortium; botanical chemistry can bias succession and stabilize/shift functional core	Saccharification and stabilization; potential sensory modulation	Similar to *Xiaoqu* but with altered acid/sugar timing and aroma modulation depending on botanical–process coupling	Added uncertainty in botanical composition and dose; risk of over-attributing “antimicrobial” effects without mechanistic evidence	Zhou et al. (2020), Zheng et al. (2025)
Pure-culture *Koji* module (*Aspergillus oryzae*)	Controlled propagation; defined inoculum; predictable enzyme production	Solid substrate with managed aeration; relatively reproducible microenvironment	“Enzyme module” dominated by a single mold; downstream fermentation depends on added yeast/bacteria	Saccharification (enzymatic) decoupled from fermentation	Highly predictable sugar release; ethanol depends on inoculated yeast; less intrinsic acid control unless added	High controllability; lower microbial variability; risks mainly operational/contamination outside defined culture	Son et al. (2018), Fei et al. (2023), Zhang J. et al. (2023)
Malt-based brewing/distilling	Malting controlled; fermentation inoculum defined	Liquid fermentation; minimal spatial heterogeneity vs. solid starters	Functional separation: enzymatic saccharification from malt and defined yeast fermentation	Saccharification and fermentation are modular and controllable	Predictable kinetics; ethanol tends toward completion unless intentionally arrested; acidity usually controlled externally	High controllability; low ecological complexity; limited relevance to *Xiaoqu*-like emergent modulation	Liu et al. (2020), Chen C. et al. (2025)

guild, functional guild; “trajectory-level,” time-resolved dynamics of sugar–acid–ethanol rather than endpoints.

## 3 Eco-evolutionary drivers of *Xiaoqu* diversity and function

### 3.1 Household domestication

*Xiaoqu* is historically sustained through repeated household and artisanal fermentation cycles that operate as an implicit, long-term selection regime. In practice, producers rarely select microbes directly; instead, they select outcomes. Starters that deliver dependable saccharification, stable fermentation behavior, and acceptable sensory profiles are more likely to be reproduced, shared, and transmitted, whereas batches associated with sluggish conversion, off-notes, or instability are discarded (Song et al., 2025). Over many cycles, this outcome-based selection favor microbial communities that can assemble and function reliably under locally typical conditions. Importantly, what is being stabilized is not a single organism, but a consortium-level capability: the coordinated coupling of saccharification, fermentation, and stabilization functions within a structured matrix. This makes “domestication” in *Xiaoqu* fundamentally different from strain domestication in industrial inoculate. It is better understood as community domestication, where repeated practice shapes interaction rules, guild balance, and resilience to environmental noise.

This framing also explains why *Xiaoqu* can maintain functional continuity despite taxonomic variability. Multiple taxa can occupy similar roles within key functional guilds, allowing functional redundancy to buffer fluctuations in membership. As long as essential guilds—amylolytic molds, fermentative yeasts, and stabilizing/acidifying bacteria—remain represented and appropriately coupled, the system can reproduce a comparable fermentation phenotype (Mao et al., 2023; Ruan et al., 2026). Repetition additionally stabilizes process cues (timing, handling, sensory indicators) that indirectly stabilize microbial assembly filters, further reinforcing reproducible architecture. Thus, household domestication does not necessarily produce a taxonomically uniform starter; rather, it selects for architectures that repeatedly generate desired metabolic trajectories, a logic directly aligned with the concepts of microbial architecture and metabolic modulation.

### 3.2 Climate as a boundary condition

Geography and climate act as boundary conditions that constrain which microbial strategies are feasible during *Xiaoqu* assembly and, consequently, which architectures are likely to emerge (Han X. et al., 2025). Temperature, humidity, and seasonality influence growth rates, enzyme expression, stress tolerance, and competitive outcomes among guilds (Yang et al., 2019; Lin et al., 2024; Han X. et al., 2025). Low-to-moderate temperature regimes—typical for *Xiaoqu* production—favor organisms capable of effective metabolism without thermophilic adaptation, while fluctuating moisture selects for taxa tolerant to shifts in water activity and to oscillating oxygen availability (Shen et al., 2025). These constraints do not simply “change the community”; they change the rules of assembly by shaping which guilds can establish early, which can persist through drying and storage, and how quickly succession proceeds during incubation.

Because *Xiaoqu* is assembled in a structured rice matrix, microenvironments amplify climatic effects. Moisture gradients, aeration constraints, and diffusion limits can create spatial niche partitioning that supports guild coexistence: aerobic molds may thrive on surfaces or within oxygen-accessible microchannels, while yeasts and bacteria exploit sugar-rich, more reduced niches generated through saccharification and local oxygen depletion. Seasonal variation can further modulate assembly by altering ambient microbial pools and by changing the feasibility of drying, which strongly influences survival and stabilization (Zhang et al., 2019; Kang et al., 2022). In this sense, climate does not merely affect “what grows”; it shapes the microenvironmental architecture within which interactions occur, thereby biasing the interaction topology that ultimately governs metabolic modulation during fermentation.

**Figure 2:**
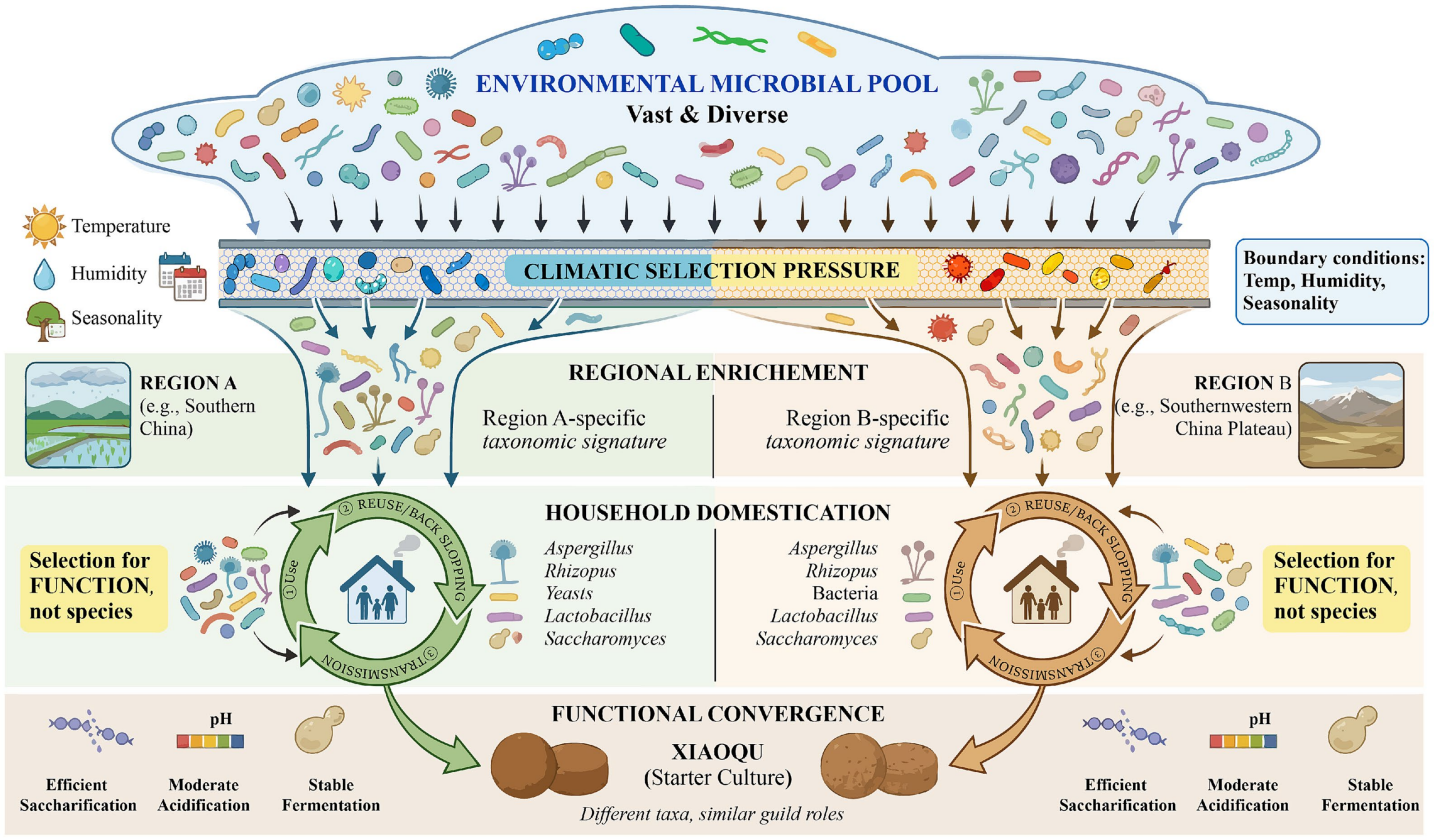
Eco-evolutionary drivers shaping *Xiaoqu* assembly and functional stability.

Taken together, the persistence and diversification of *Xiaoqu* can be understood as the outcome of eco-evolutionary filtering operating at the level of community performance rather than taxonomic identity. Repeated household fermentation cycles implicitly select for starter batches that deliver reliable saccharification and acceptable sensory outcomes, favoring consortium-level robustness over the success of individual species. Geography and climate further delimit the space of feasible physiological strategies during starter making, imposing boundary conditions through temperature, humidity, and seasonality. Within these constraints, *Xiaoqu* communities diverge in membership yet often converge in function, particularly when deployed toward similar glutinous rice wine phenotypes. This coexistence of regional microbial signatures with conserved fermentation trajectories indicates that stability is encoded in guild structure, interaction constraints, and redundancy rather than in fixed taxa lists. Regional variation therefore serves not as noise but as a natural experiment revealing how different assemblies can realize the same functional architecture under shared ecological pressures (Figure 2).

### 3.3 Regional signatures and functional convergence

Regional divergence in community membership is widely observed in *Xiaoqu*, yet fermentation outcomes can converge when starters are used toward similar glutinous rice wine goals. This coexistence of taxonomic divergence and functional convergence is highly informative: it suggests that stability is encoded at the level of guild structure and interaction constraints, rather than strict species identity. Regionality therefore functions as a natural experiment. Different climates, ambient microbial reservoirs, and micro-practices generate distinct community fingerprints, but similar fermentation phenotypes imply that multiple ecological solutions can converge on comparable architectures—each maintaining the coupling needed to produce rapid saccharification, controlled acidification, and restrained ethanol accumulation (Kim et al., 2024; Chen C. et al., 2025).

This convergence perspective also reframes what should be conserved across regions. Instead of searching exclusively for a universal taxonomic “core,” it is often more productive to identify a conserved functional core: recurring representation of saccharification, fermentation, and stabilization roles, and conserved trajectory-level features in sugar–acid–ethanol dynamics (Chen et al., 2021b; Liu et al., 2024; Tian et al., 2025). The analytical implication is clear. Comparative synthesis should emphasize shared ecological metrics—guild balance, redundancy, core–satellite patterns, and interaction proxies—alongside shared functional readouts, particularly time-resolved trajectories rather than end-point chemistry. Taxa lists remain useful, but they are difficult to compare across pipelines and often overstate differences that may be functionally irrelevant. By focusing on architecture-level descriptors and trajectory-level phenotypes, regional variation becomes a tool for inference: it reveals which features of *Xiaoqu* are flexible (membership) and which are constrained (organization and modulation), thereby clarifying how eco-evolutionary drivers shape architectures that reliably deliver glutinous rice wine fermentation performance.

## 4 Process-shaped assembly of *Xiaoqu* microbial architecture

### 4.1 The matrix as habitat

*Xiaoqu* production is most usefully interpreted as habitat engineering, in which raw materials and handling choices construct the physical–chemical landscape that determines microbial recruitment, survival, and interaction. Rice-derived substrates provide a carbohydrate-rich matrix with limited readily accessible nitrogen and micronutrients, favoring organisms that can rapidly mobilize resources via extracellular enzymes and exploit heterogeneous solid substrates (Hong et al., 2023). In this setting, molds gain an early ecological advantage because hyphal growth enables penetration of the matrix, spatial exploration, and sustained enzyme secretion, thereby initiating starch depolymerization and reshaping the local resource economy. Critically, the matrix is not a passive carrier of microbes; it is an active determinant of microbial architecture because it defines diffusion constraints, local oxygen availability, and the micro-niche structure within which guilds partition space and function.

Physical properties of the matrix strongly influence assembly outcomes. Particle size and hydration govern effective surface area and connectivity between micro-niches; porosity and cohesion determine oxygen diffusion and water distribution; and mechanical structure influences whether hyphal networks can establish a scaffold that supports spatially organized coexistence. A dense, poorly aerated matrix may suppress mold growth and favor facultative anaerobes, shifting the balance toward acidification-dominated trajectories. Conversely, a moderately porous matrix can support mold-driven saccharification while enabling yeasts and bacteria to occupy complementary niches shaped by sugar gradients and oxygen limitation (Bal et al., 2017; Kim and Seo, 2021; Liu et al., 2021; Cao et al., 2022; Lee et al., 2023). These structural dependencies provide a mechanistic route from “how the starter is made” to “what kind of architecture emerges,” and they directly foreshadow downstream metabolic modulation in glutinous rice wine fermentations, where saccharification–acidification–fermentation coupling is trajectory-defining.

Where botanicals are used, they should be treated as modifiers of the selection landscape rather than deterministic antimicrobials. Botanical additives introduce complex phytochemical mixtures that can impose chemical constraints, bias competitive hierarchies, and alter succession timing (Zhang Y. et al., 2023; Geng et al., 2024a; Geng et al., 2024b; Zhang et al., 2025a). However, their effects are unlikely to be uniform across batches because they interact with processing variables (hydration, incubation, drying rate) and evolving microenvironments (pH, redox, metabolite accumulation) (Shen et al., 2025). A more informative framing is therefore process–phytochemical selection, which is proposed here as a literature-derived conceptual synthesis and testable ecological hypothesis rather than as a fully validated mechanistic model. In this framework, botanical chemistry is considered one layer of selection embedded within a broader habitat and process context. Phytochemicals may shape microbial assembly by constraining sensitive taxa, shifting competitive hierarchies, or altering succession timing, but these effects are expected to depend on processing variables such as hydration, incubation temperature, drying rate, oxygen availability, pH, redox state, and metabolite accumulation. Thus, botanicals should not be interpreted as deterministic antimicrobials; instead, they may interact with process conditions to bias assembly trajectories and contribute to the stabilization of functional architectures (Subramaniyan et al., 2016; Geng et al., 2024a; Wang Y. et al., 2024; Shen et al., 2025; Xiong Z. et al., 2025). Direct validation of this framework will require controlled starter-making experiments combined with phytochemical profiling, strain-level community analysis, transcriptomics, metabolomics, and synthetic-community reconstruction.

### 4.2 Steps as selective filters

If the matrix defines the habitat, the production steps define the filters that determine which microbes can establish and how the consortium is organized. Mixing does more than homogenize ingredients; it seeds micro-heterogeneity in hydration and nutrient distribution and disperses microbes introduced from raw materials, tools, and ambient environments. These initial conditions can influence early colonization advantages, particularly for molds that exploit localized moisture and oxygen access. Shaping transforms the substrate into discrete units and thereby sets surface-to-volume ratios, internal aeration, and the extent to which heat and moisture are retained (Cao et al., 2022; Xia et al., 2023; Song et al., 2025; Peng et al., 2026b). These geometric features strongly influence whether aerobic mold growth can be sustained and how rapidly oxygen becomes limiting in internal micro-niches.

Incubation functions as the primary assembly stage, were temperature and humidity tune guild balance and interaction topology (Zong et al., 2026). Under the low-to-moderate temperatures typical of *Xiaoqu*, incubation conditions favor organisms capable of efficient enzyme production and growth without thermophilic adaptation, while also selecting for tolerance to variability. Because incubation is often loosely controlled, microbes experience fluctuating rather than fixed conditions, which can select for physiological robustness and stabilize architectures that remain functional under noise. The timing of guild establishment during incubation is especially important: early mold success can generate rapid saccharification and scaffold formation, whereas early bacterial dominance can accelerate acidification and suppress saccharifying activity, potentially redirecting the system toward fewer desirable trajectories (Jiang et al., 2026).

Drying and storage are frequently described as preservation steps, but they also act as ecological filters that shape viability, enzyme persistence, and the “frozen” succession state of the starter (Jiang et al., 2020; Wang C. X. et al., 2020; Zhao et al., 2022; Chen L. C. et al., 2023). Drying lowers water activity and arrests rapid growth, limiting overexpansion of opportunists and stabilizing the starter for subsequent use. Yet drying rate and conditions can differentially affect guild survival and enzyme integrity, thereby altering how the architecture reactivates during fermentation. Storage conditions further modulate recovery and drift, influencing which guilds dominate early upon inoculation into a glutinous rice substrate (Yu et al., 2021b). Together, these production steps act as coordinated ecological filters that shape microbial assembly, determine which architectural features are retained, and ultimately govern how reliably the consortium reproduces a desired fermentation trajectory under ambient conditions.

**Figure 3:**
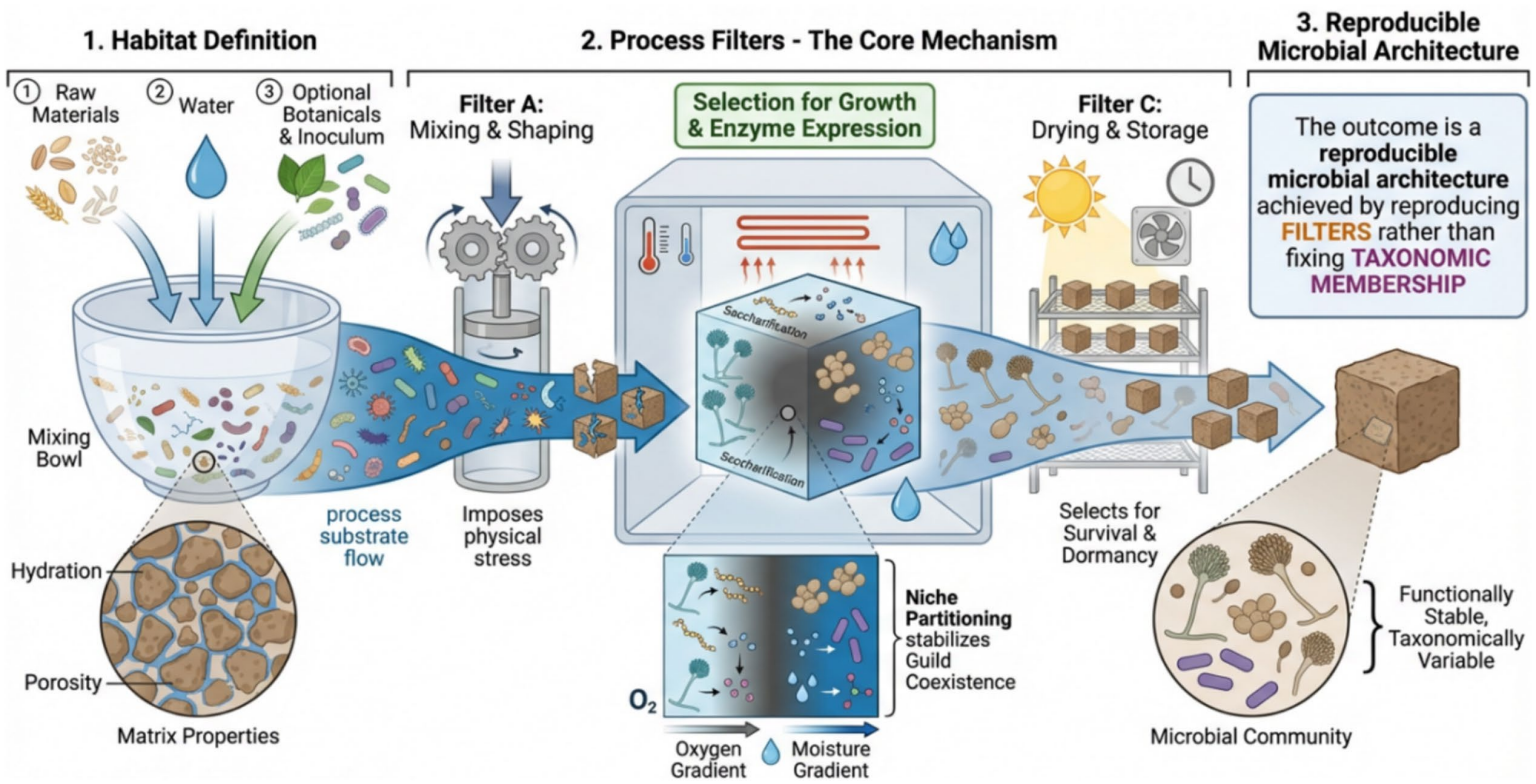
Processing steps as ecological filters shaping *Xiaoqu* microbial assembly.

The assembly of *Xiaoqu* does not depend on a defined inoculum but on the repeated imposition of process-derived ecological filters. Mixing disperses microorganisms from raw materials and local environments while seeding micro-heterogeneity within the rice matrix (Song et al., 2025). Shaping defines surface-to-volume ratios, internal aeration paths, and diffusion distances, thereby constraining oxygen availability and moisture distribution. Incubation constitutes the primary assembly phase, during which temperature and humidity selectively favor organisms capable of low-temperature growth, extracellular enzyme secretion, and tolerance to fluctuating water activity. Drying arrests succession and stabilizes the starter, while subsequent storage conditions influence survival, recovery, and early-stage dominance upon reuse (Shen et al., 2025; Song et al., 2025). Collectively, these steps generate structured microenvironments characterized by gradients in temperature, moisture, oxygen, and metabolites, which repeatedly assemble similar guild configurations despite variability in taxonomic membership (see Figure 3). Taken together, mixing, shaping, incubation, drying, and storage do not merely constitute a production workflow but function collectively to shape how *Xiaoqu* microbial communities are assembled and stabilized under ambient conditions.

### 4.3 Key parameters and ecological mechanisms

The ecological power of *Xiaoqu* processing lies in the fact that key parameters generate gradients and microenvironments, rather than uniform conditions. Temperature governs growth rates and enzyme expression, but in structured matrices it can also interact with moisture and aeration to shape spatial heterogeneity. Moisture and water activity regulate diffusion, connectivity between niches, and the feasibility of sustained metabolic activity across the starter unit. Oxygen availability—strongly influenced by porosity and shaping—controls mold feasibility and modulates whether yeasts and bacteria operate in more respiratory or fermentative modes. These parameters jointly influence the architecture by determining whether guilds can coexist through niche partitioning, whether competition becomes exclusionary, and whether interactions are stabilized through feedback constraints.

Two dynamic mechanisms are particularly important because they link assembly directly to downstream fermentation modulation. The first is resource-release dynamics, driven primarily by mold saccharification (Liu et al., 2019; Sakandar et al., 2020; Olee et al., 2022). The rate and spatial distribution of sugar release create a time-varying substrate landscape that governs yeast expansion and bacterial competitiveness. If sugar release is rapid but poorly coupled to uptake, sugars may accumulate and shift the system toward opportunistic trajectories; if sugar release is synchronized with consumption, the system can sustain stable fermentation while supporting sweetness retention. The second is acidification dynamics, mediated largely by bacteria. pH kinetics can act as an internal control layer that stabilizes succession by suppressing undesired microbes and tuning enzyme activity, but excessive or premature acidification can inhibit saccharification and disrupt mold–yeast coupling (Wang C. X. et al., 2020; Zhu et al., 2023). These two dynamics—sugar release and pH kinetics—operate as feedback signals that constrain succession and help maintain coupling. They are therefore central mechanistic bridges between microbial architecture (who can establish and interact) and metabolic modulation (how sugar–acid–ethanol trajectories unfold).

### 4.4 From filters to architecture

Because inoculation in *Xiaoqu* is not strictly defined, reproducibility cannot be achieved by fixing community membership in the manner of industrial starters (Cheng et al., 2011; Liu et al., 2021; Huang et al., 2023). Instead, reproducibility arises from reproducing the filters—the characteristic ranges of matrix properties and processing conditions that repeatedly assemble a functionally organized consortium. Architecture emerges when the process and environment consistently generate guild structure, redundancy, and interaction constraints that channel succession toward productive states. This perspective clarifies why *Xiaoqu* can exhibit taxonomic variability yet retain functional stability: different taxa can occupy equivalent guild roles as long as the filters preserve the interaction topology and coupling regime needed for desired trajectories.

This reframing has direct implications for modernization and starter improvement. The goal is not simply to replace *Xiaoqu* with defined strains, which may stabilize membership but fail to recreate architecture-driven modulation. Rather, the goal is to identify which filters are necessary and sufficient to assemble desirable architectures and to determine how those filters can be reproduced—or strategically tuned—to meet modern expectations for safety and consistency without sacrificing functional robustness. Operationally, this suggests shifting attention from “standardizing species” to “standardizing environments and trajectories”: specifying measurable process parameters (moisture, porosity, incubation regimes), tracking architecture-relevant indicators (guild balance, enzyme activities), and validating performance through time-resolved sugar–acid–ethanol dynamics in glutinous rice wine fermentations. In doing so, *Xiaoqu* becomes a model for controllable natural assembly—one where ecological filtering, rather than inoculum definition, is the primary lever for engineering microbial architecture and its emergent metabolic modulation.

## 5 Architecture to trajectory: interactions and metabolic modulation in fermentation

### 5.1 Guild organization and interaction topology

The functional identity of *Xiaoqu* is most clearly expressed during fermentation, where its microbial architecture becomes observable as coordinated conversion, stabilization, and sensory modulation. Rather than behaving as a collection of independent species, *Xiaoqu* operates as an organized consortium in which molds, yeasts, and bacteria form coupled modules. Amylolytic molds initiate starch deconstruction and often dominate early resource mobilization by secreting extracellular hydrolases; in doing so, they define the primary substrate landscape that later constrains yeast and bacterial metabolism. Fermentative yeasts convert the released sugars into ethanol and a broad range of secondary metabolites, yet their flux is not simply determined by sugar availability: it is shaped by spatial access, pH, redox conditions, and inhibitory environments constructed by other guilds. Bacterial guilds—frequently including LAB—further regulate the system by modulating pH, shaping succession, and contributing organic acids and other metabolites that influence both microbial stability and sensory balance. The decisive feature is therefore not “who is present,” but how functions are wired: cross-feeding links sugar release to fermentation; pH-mediated regulation couples acidification to enzyme activity and competitiveness; and competition for oxygen and micronutrients structures succession timing and niche occupancy (Jiang et al., 2026).

Recent advances in *Xiaoqu*-related fermentation studies further support this guild- and interaction-based interpretation. For example, core microbiota identification and synthetic-community reconstruction have begun to move *Xiaoqu* research from correlative community profiling toward experimental testing of functional guilds and their contributions to fermentation performance. Integrated metagenomic and metabolomic analyses also provide a way to link microbial succession with enzyme activity, substrate conversion, and flavor-compound formation, thereby clarifying how community structure is translated into metabolic trajectories. These approaches are particularly valuable for distinguishing taxa that are merely abundant from those that are functionally important within the consortium (Wang H. et al., 2024; Han M. et al., 2025).

Within this interaction topology, mold–yeast coupling often constitutes the architectural backbone that connects saccharification to fermentation performance. The timing and localization of sugar release determine whether yeast uptake tracks saccharification or whether sugars accumulate and redirect community dynamics. Hyphal growth can increase habitat heterogeneity by creating diffusion pathways and oxygen micro-gradients, supporting niche partitioning and stabilizing coexistence. Conversely, insufficient aeration or excessive moisture may suppress mold performance, weakening saccharification and shifting the balance toward bacterial dominance (Morales et al., 2015; Qu et al., 2022). Importantly, yeast attenuation in glutinous rice wine contexts should be viewed as a consortium property rather than a yeast-only parameter. Yeasts respond to sugar spectra, oxygen gradients, pH trajectories, and inhibitory metabolites, meaning that “fermentative strength” emerges from the microenvironment produced by mold-driven resource release and bacterial regulation. This provides a mechanistic basis for a defining phenotype of many glutinous rice wine styles: sweetness retention with restrained ethanol accumulation, which can arise when saccharification, acidification, and yeast flux are coupled within a constrained regime rather than maximized independently.

Bacterial guilds integrate into this backbone by acting as both stabilizers and modulators. Moderate acidification can suppress opportunists, reduce ecological noise, and stabilize succession, thereby protecting the coupling between saccharification and fermentation. However, excessive or poorly timed acidification can suppress enzyme activity and disrupt mold–yeast coupling by shifting the system into a low-pH state that penalizes saccharification and constrains yeast metabolism (Lin et al., 2022; Du et al., 2023; Zhang et al., 2025b; Zong et al., 2026). LAB therefore function as regulatory actors embedded in feedback loops, not as passive cohabitants. Their influence on yeast stress physiology and aroma-related metabolism further integrates bacteria into architecture-driven outcomes. Taken together, these interactions explain why *Xiaoqu* can be taxonomically variable yet functionally stable: stability emerges from two complementary mechanisms—functional redundancy within guilds (including enzymatic overlap, niche overlap, and alternative metabolic pathways) and constraint-based stability imposed by microenvironmental gradients and feedback control. This also clarifies why replacing *Xiaoqu* with a single culture often fails: a single organism can replicate a function, but it rarely reconstructs the interaction topology that produces the desired trajectory.

The outcome of process-shaped assembly is not a random mixture of organisms but a reproducible microbial architecture organized around interacting functional guilds. In *Xiaoqu*, this architecture is most clearly expressed through the partitioning and coupling of amylolytic molds, fermentative yeasts, and bacterial modulators. Molds constitute the primary saccharification module, supplying extracellular enzymes and, through hyphal growth, structuring microhabitats that constrain diffusion and niche access. Yeasts occupy a secondary but tightly coupled module, converting released sugars into ethanol and secondary metabolites under constraints imposed by pH, nutrient availability, and inhibitory by-products. Bacterial guilds, frequently including LAB, function as regulatory components that modulate acidification, succession, and stability rather than as passive cohabitants (Tian et al., 2022; Zhu et al., 2026).

**Figure 4:**
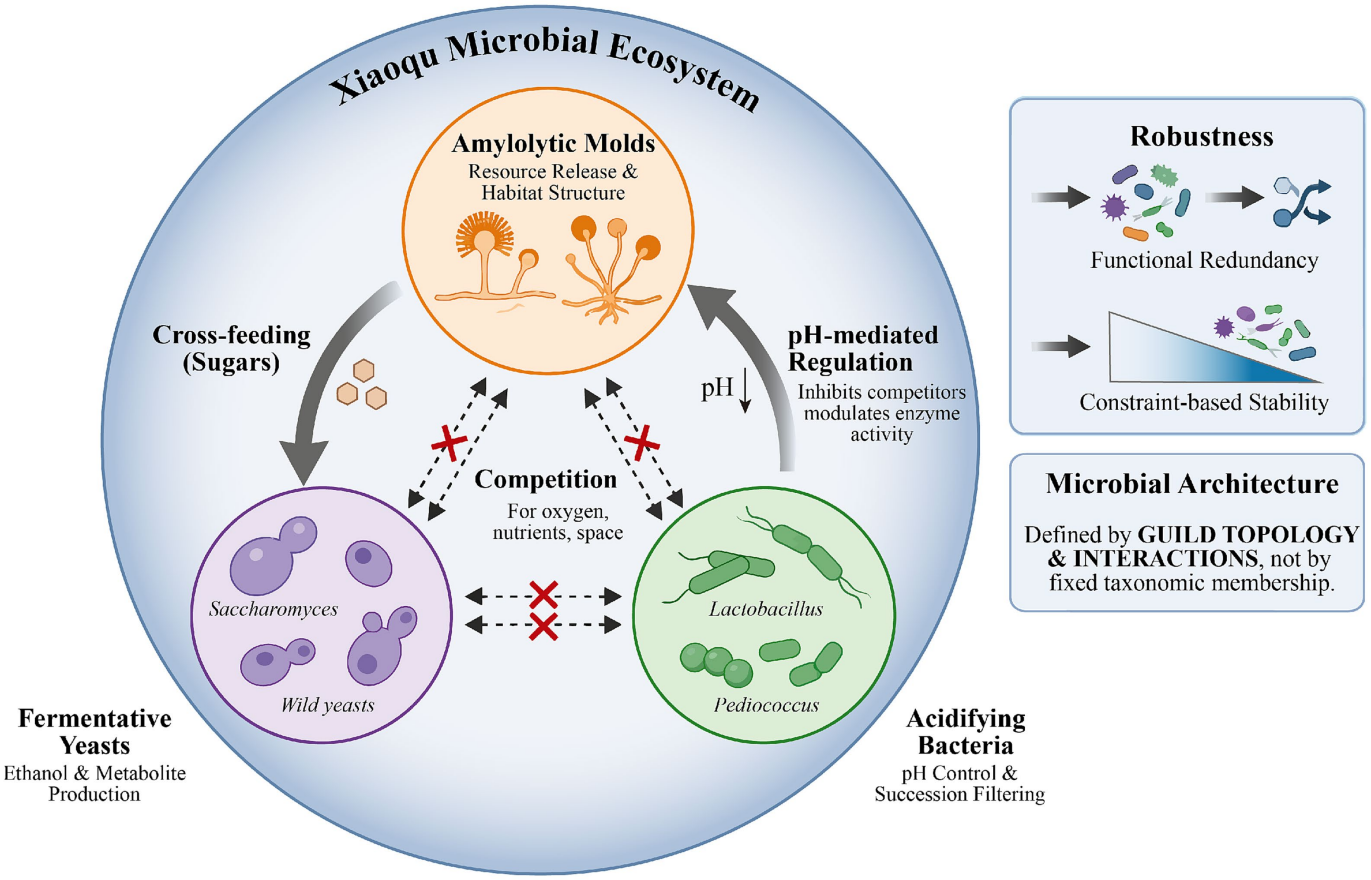
Microbial architecture of *Xiaoqu* expressed as functional guilds and interaction topology.

Crucially, system performance emerges from the topology of interactions among these guilds rather than from the dominance of any single group. Cross-feeding links sugar release to fermentative flux, pH-mediated regulation couples’ bacterial activity to enzyme performance and competitiveness, and competition for oxygen and micronutrients shapes temporal succession (Zhu et al., 2026). Functional redundancy within each guild buffers taxonomic variability, while interaction constraints stabilize consortium-level behavior. This guild-based organization and interaction topology define the microbial architecture of *Xiaoqu* and provide the structural basis for its functional robustness across variable conditions (Figure 4).

### 5.2 Metabolic modulation as trajectories

Metabolic modulation in *Xiaoqu*-mediated fermentation is best described as the coordinated evolution of key state variables over time. Sugars, pH, and ethanol are not merely end products; they function as internal control signals that regulate enzyme performance, microbial competitiveness, and interaction outcomes (Zong et al., 2026). A productive fermentation can be conceptualized as remaining within a desirable region of “trajectory space,” characterized by three coupled features: (i) sugar release is rapid but not chronically decoupled from uptake; (ii) pH declines to stabilizing levels without collapsing saccharification capacity; and (iii) ethanol increases but plateaus before complete sugar depletion in sweet-style systems. This trajectory framing is essential because end-point analyses obscure timing and coupling—precisely the dimensions where microbial architecture exerts control and where glutinous rice wine phenotypes are determined.

Trajectory thinking also clarifies why *Xiaoqu* can be robust under weak control. Robustness does not necessarily mean insensitivity to perturbation; rather, it implies that the system contains self-regulating feedbacks that prevent runaway dynamics and constrain succession. Rising sugars reshape osmotic and energetic constraints and can alter competitive hierarchies; acidification filters opportunists and shifts enzyme optima; ethanol and organic acids contribute inhibition that can dampen excessive flux (Miao et al., 2022; Luo et al., 2024). These feedbacks are consortium-derived: they depend on the balance among guilds and on the interaction topology that couples resource release to consumption and stabilization. Metabolic modulation is therefore not “tuning” applied from outside; it is an emergent property of organized consortia embedded in structured microenvironments. This conceptualization naturally bridges microbial architecture to practical performance, because it translates community organization into measurable, time-resolved outcomes relevant to glutinous rice wine fermentation.

### 5.3 Sugar–acid–ethanol coupling and restraint

Sugar landscapes. Rapid saccharification generates heterogeneous and time-varying sugar environments. In solid or semi-solid contexts, diffusion limits and micro-niches create localized sugar enrichment, which affects both yeast access and microbial competition. Rather than a single pool of fermentable substrate, the fermentation matrix can contain gradients and pockets where sugars are released and consumed at different rates. Mixed sugar spectra—including oligosaccharides and partially hydrolyzed fragments—can further support substrate partitioning, enabling co-existence among yeast and bacterial lineages with different uptake preferences and thereby stabilizing guild balance. In this context, modulation is expressed not only in total reducing sugars but in sugar kinetics and profiles: the timing of peak sugar release, the persistence of non-glucose fractions, and the coupling between sugar availability and yeast flux (Horváth et al., 2020; Yu et al., 2023b; Tian et al., 2025).

Acid dynamics. Controlled acidification is a system-level lever that shapes both ecology and chemistry (Chen L. et al., 2025; Tian et al., 2025; Zhu et al., 2026). pH trajectories regulate enzyme activity, shift competitive hierarchies, and modulate sensory balance by shaping organic acid profiles and sweetness perception. Timing is decisive. Early acidification can stabilize succession by suppressing opportunists and reducing ecological noise, thereby protecting saccharification–fermentation coupling. Excessive or prematurely rapid acidification can suppress mold-driven enzyme function and reduce sugar release, effectively breaking the coupling that sustains productive trajectories. Spatially structured acidification may also contribute to stability by supporting niche partitioning, meaning bulk pH measurements can underrepresent microenvironment regulation that is critical to interaction topology. Thus, acidification should be interpreted as a dynamic control layer, not a simple output variable.

Ethanol restraint. Many glutinous rice wine fermentations show restrained ethanol accumulation despite effective saccharification, highlighting the distinction between potential fermentability and realized metabolic flux. Ethanol restraint (Ji et al., 2025) should therefore not be treated as a single phenomenon, because low ethanol accumulation may arise from two mechanistically distinct scenarios: (i) unrealized metabolic potential (incomplete fermentation) and (ii) metabolic rerouting under active system constraints.

In the first scenario, ethanol remains low because fermentable substrates are not fully converted into ethanol despite saccharification. Such incomplete fermentation may result from moderated access to sugars caused by spatial structure and temporal release patterns, such that released carbohydrates are not equally accessible for yeast utilization. In addition, micronutrient limitation typical of rice matrices, inhibitory metabolite accumulation (including ethanol and organic acids), and unfavorable pH conditions can restrict yeast biomass expansion and fermentation capacity, generating early plateau behavior before complete substrate consumption (Yang et al., 2018; Ji et al., 2025).

In the second scenario, low ethanol reflects metabolic rerouting rather than incomplete utilization. Here, carbon flux is actively redistributed toward non-ethanol products, including organic acids, glycerol, polyols, and other secondary metabolites produced by non-*Saccharomyces* yeasts and bacteria. Non-*Saccharomyces* yeasts may play particularly important roles in this rerouting process. Compared with *Saccharomyces cerevisiae*, many non-*Saccharomyces* yeasts exhibit lower ethanol yields, distinct substrate preferences, and broader secondary-metabolite production profiles, thereby contributing to glycerol formation, organic acid accumulation, aroma diversification, and sweetness retention (Deng et al., 2025; Li et al., 2025; Amorim et al., 2026). Beyond direct metabolite production, these yeasts may also influence fermentation trajectories through cross-feeding, micronutrient competition, and modulation of local stress environments within the consortium. Acidification, feedback regulation, and stress responses may further redirect yeast metabolism from growth-associated ethanol production toward maintenance and secondary metabolism (Hu et al., 2025). Under these conditions, low ethanol becomes an emergent property of community organization and feedback control rather than evidence of fermentation failure.

Importantly, ethanol restraint in sweet-style glutinous rice wine may therefore represent either incomplete fermentation or an adaptive metabolic phenotype. Distinguishing these mechanisms requires trajectory-level evaluation, including residual sugar utilization, ethanol yield relative to consumed carbon, carbon partitioning into non-ethanol metabolites, and temporal coupling between saccharification and fermentation.

Molecular regulation likely underlies these trajectory-level phenotypes. Beyond trajectory-level observations, these coupling regimes likely reflect underlying molecular and regulatory interactions among guilds. Multi-omics studies increasingly suggest that sugar-acid-ethanol trajectories are shaped not only by substrate availability but also by coordinated regulation of carbohydrate utilization, stress responses, redox balance, and secondary metabolism across interacting microorganisms (Tian et al., 2025; Yang et al., 2025). For example, acidification by LAB may alter yeast transcriptional responses associated with glycolysis, ethanol tolerance, and maintenance metabolism, thereby influencing whether carbon flux is directed toward ethanol production or rerouted into organic acids, glycerol, polyols, and aroma-associated metabolites. Similarly, fungal hydrolytic activity and temporal sugar release may regulate competitive access to fermentable substrates, indirectly shaping yeast metabolic states and succession dynamics.

Cross-feeding interactions may further stabilize coupled metabolic trajectories (Zhu et al., 2026). Organic acids, amino acids, peptides, vitamins, and partially hydrolyzed carbohydrates generated by molds or bacteria can function not only as metabolites but also as ecological signals and regulatory substrates influencing growth and pathway activation in neighboring guilds. From this perspective, sugar-acid-ethanol coupling may be interpreted as an emergent systems-level phenotype arising from interacting metabolic networks rather than from independent activities of isolated taxa. Future integration of strain-level metagenomics, transcriptomics, metabolomics, and flux-oriented analyses will be essential for identifying which regulatory interactions reproducibly govern carbon partitioning, fermentation restraint, and sweetness retention in *Xiaoqu*-mediated glutinous rice wine fermentations.

Operationally, these couplings imply that metabolic modulation should be assessed by trajectory metrics rather than static end points.

### 5.4 Implications for glutinous rice wine performance

The architecture-to-trajectory framework provides a mechanistic explanation for why *Xiaoqu* can deliver functional robustness and sensory consistency under variable conditions: guild redundancy buffers membership variation, while interaction constraints and microenvironmental gradients stabilize trajectories. This logic also clarifies why simple substitution strategies often underperform. Defined inoculate can stabilize individual modules—such as ethanol production or acidification—but unless they reconstruct the interaction topology and trajectory control inherent to *Xiaoqu*, they may fail to reproduce sweetness retention, moderated acidification, and ethanol restraint simultaneously. The core challenge is therefore not to maximize individual functions, but to reproduce the coupling regime that generates the desired sugar–acid–ethanol trajectory (Wu et al., 2024).

To operationalize trajectory-based analysis, future studies should adopt time-resolved sampling strategies rather than relying only on endpoint comparisons. For *Xiaoqu* starter-making, a minimum sampling framework may include T0-T4 (starter trajectory stages), where T denotes stages during starter production: T0, raw material mixing/inoculation; T1, early colonization; T2, active microbial growth and saccharification; T3, maturation or drying; and T4, storage or pre-use starter (Wang Z. M. et al., 2020; Xia et al., 2023). For glutinous rice wine fermentation, a minimum framework may include F0-F4 (fermentation trajectory stages), where F denotes stages during wine fermentation: F0, rice inoculation; F1, early saccharification; F2, active alcoholic fermentation; F3, acidification and flavor development; and F4, final product. Denser sampling during early succession (T1-T2) and active fermentation (F1-F2) is recommended because microbial growth, enzyme production, interaction restructuring, and carbon flux change rapidly during these windows (Peng et al., 2025).

Standard dynamic metrics should include microbial indicators, such as community turnover, alpha- and beta-diversity trajectories, succession directionality, time-lagged associations, network rewiring, and keystone-taxon persistence; functional indicators, including guild-level abundance dynamics and enzyme activity curves; and metabolic indicators, including substrate consumption rates, ethanol yield per consumed sugar, organic acid and polyol accumulation, and volatile compound formation rates (Yu et al., 2023a; Chang et al., 2026). Such metrics enable differentiation between endpoint similarity and trajectory similarity and facilitate linking microbial architecture to fermentation phenotypes.

A rational modernization pathway is thus “architecture-preserving control.” Rather than targeting strict taxonomic standardization, this approach aims to preserve guild functions, redundancy, and feedback structure while improving reproducibility and safety through measurable filters, targeted stabilization, and validated trajectory endpoints. Practically, this suggests shifting quality control from species lists to architecture- and trajectory-relevant indicators: enzyme activities linked to saccharification timing, pH kinetics that stabilize without suppressing saccharification, and ethanol trajectories that plateau appropriately for sweet-style products. It also supports the concept of modular starter strategies—whether fully traditional, hybrid, or minimal consortia—so long as they preserve interaction topology and deliver the required metabolic modulation. Framed in this way, *Xiaoqu* becomes not only a culturally important starter but also a scientifically tractable model for how process-shaped microbial architectures can be leveraged to achieve stable, desirable fermentation phenotypes in glutinous rice wine systems under weak control.

**Figure 5:**
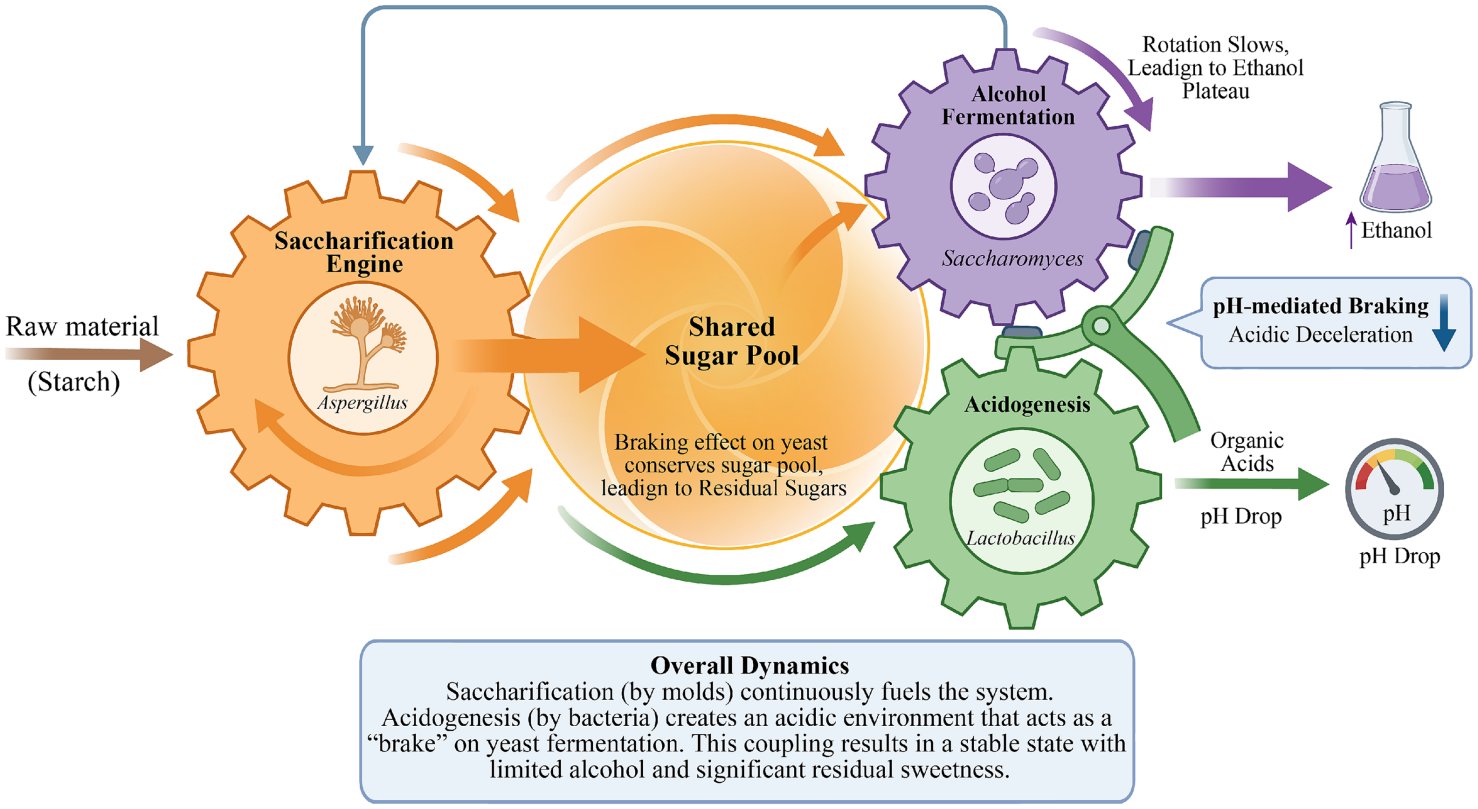
Metabolic modulation in *Xiaoqu* fermentation through coupled sugar, acid, and ethanol trajectories.

The functional consequences of microbial architecture in *Xiaoqu* are most clearly expressed as metabolic modulation along time-resolved fermentation trajectories. In *Xiaoqu*-mediated systems, sugars, acids, and ethanol are not merely end products but dynamic state variables that both reflect and regulate microbial activity (Wang et al., 2025; Wei et al., 2025). Rapid saccharification generates evolving sugar landscapes that define yeast access and competitive balance, while concurrent acidification modulates enzyme activity, microbial succession, and sensory equilibrium. Ethanol accumulation, in turn, feeds back through inhibitory and resource constraints, shaping late-stage fluxes. The defining feature is not any single rate or endpoint, but the coordinated coupling of these variables over time, which channels fermentation toward phenotypes characterized by sweetness retention, moderated acidity, and restrained ethanol accumulation (see Figure 5). Together, these coupled trajectories translate microbial architecture into robust fermentation performance under variable conditions.

Reframing *Xiaoqu* as a process-shaped microbial architecture enables a shift from empirical replication to architecture-preserving control. Rather than replacing traditional starters with simplified pure cultures, controllability can be achieved by identifying and stabilizing the ecological filters, guild structures, and interaction constraints that generate desired metabolic trajectories. In this view, modernization does not require eliminating diversity but channeling it through measurable and reproducible parameters that maintain saccharification–acidification–fermentation coupling.

**Figure 6:**
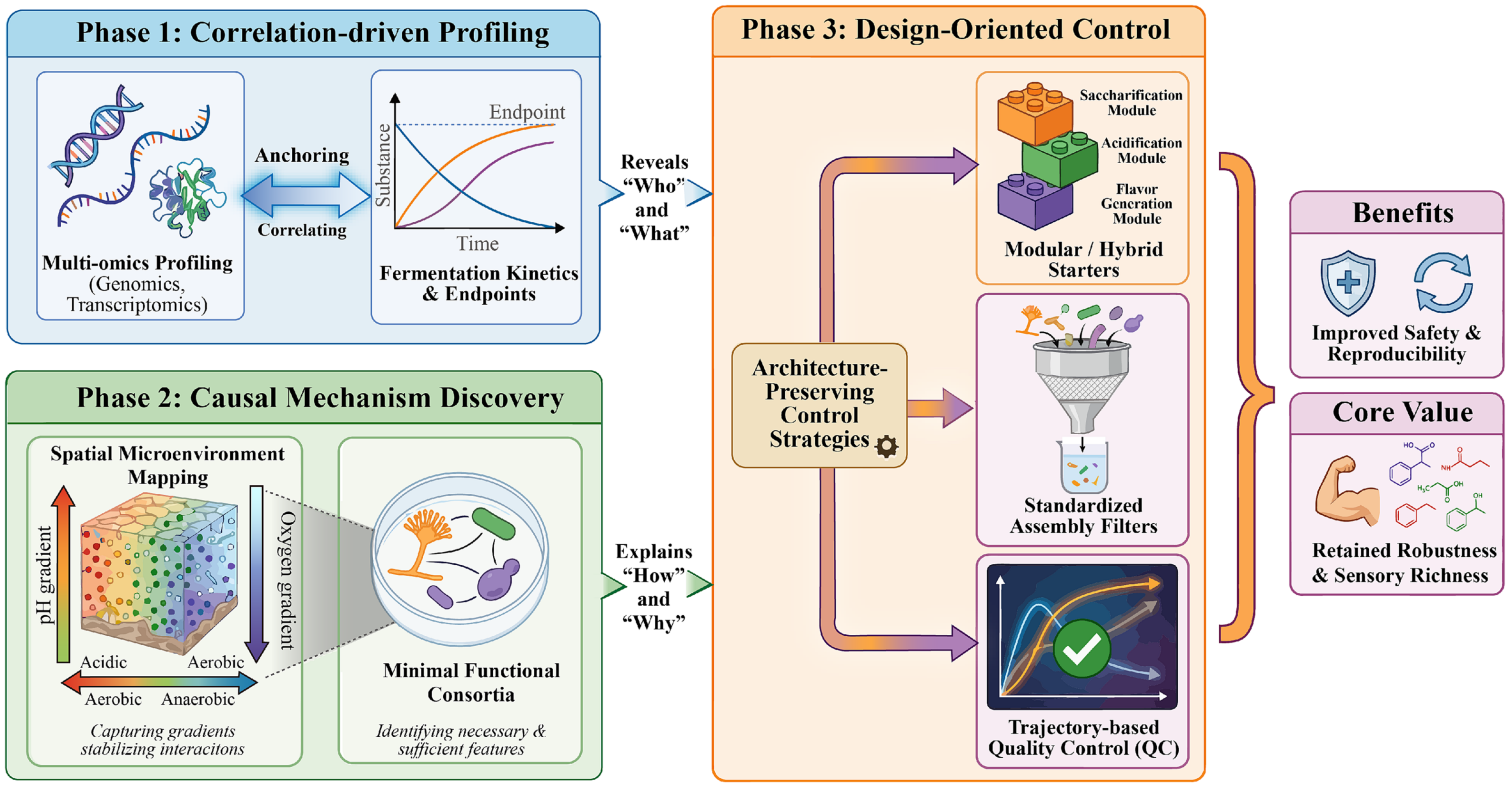
Translating microbial architecture into controllable starter design strategies.

Such a framework supports modular and hybrid starter strategies in which specific functions—such as saccharification capacity, acidification kinetics, or yeast attenuation—are selectively stabilized without dismantling consortium-level regulation. Trajectory-based metrics, including sugar peak timing, pH kinetics, and ethanol plateau behavior, provide operational endpoints for validating whether architectural integrity is preserved. By linking microbial architecture to controllable process features and performance criteria, *Xiaoqu* becomes a model system for designing robust, low-input fermentation starters that reconcile traditional complexity with modern demands for safety, consistency, and scalability (see Figure 6). Translating microbial architecture into actionable control requires metrics that capture not only end-point chemistry but also the dynamics and coupling of fermentation trajectories. Representative operational metrics linking guild organization to metabolic modulation are summarized in Table 2.

**Table 2:** Operational metrics linking microbial architecture to metabolic modulation in *Xiaoqu*-mediated glutinous rice wine fermentation.

Conceptual element	What to quantify	Recommended readouts	Trajectory metric examples (operational)	Mechanistic interpretation	Common pitfalls
Guild balance (architecture)	Relative contributions of molds/yeasts/bacteria	Amplicon/shotgun and absolute quantification (qPCR/spike-in); enzyme assays	Guild balance index (normalized mold:yeast: LAB proportions over time)	Identifies whether the system is in a saccharification-led, fermentation-led, or acidification-led regime	Relative abundance without absolute scaling; inferring function from taxonomy alone
Redundancy and robustness (architecture)	Functional redundancy within guilds; stability of performance under perturbation	Functional gene profiles; replicate fermentations under mild parameter shifts	Robustness score (variance of trajectory metrics across replicates/conditions)	Explains why membership varies yet phenotype converges; supports “function over membership”	Confounding by uncontrolled environment; “robustness” claimed without perturbation design
Interaction topology (architecture)	Cross-feeding and pH-mediated coupling strength	Time-series multi-omics and kinetics; correlation constrained by temporal directionality	Coupling ratio = (rate of sugar consumption)/(rate of sugar release) over time	Quantifies synchronization of saccharification and fermentation; low coupling predicts sugar accumulation and ecological drift	Cross-sectional correlations misread as interactions; ignoring time lags
Sugar landscapes (modulation)	Sugar profiles and their timing (not only total reducing sugar)	HPLC/LC; enzymatic sugar panels; sampling across time	Sugar peak timing (t_peak); residual sweetness window (duration above sweetness threshold)	Links mold enzyme activity and diffusion constraints to yeast access and sweetness retention	Endpoint-only sugar reporting; sampling too sparse to resolve peaks/plateaus
Acid dynamics (modulation)	pH kinetics and organic acid trajectories	pH time series; organic acids (HPLC/LC); microelectrode where feasible	pH half-time (t_½ pH drop); acid-to-sugar timing offset	Interprets acidification as control layer that stabilizes succession and tunes enzyme optima	Bulk pH masks microenvironments; attributing acid solely to LAB without flux evidence
Ethanol restraint (modulation)	Plateau behavior and incomplete attenuation in sweet-style systems	Ethanol time series; biomass proxies; redox/cofactor indicators	Ethanol plateau index (EPI) = 1 − (ΔEtOH late/ΔEtOH early)	Captures early acceleration but late slowing/plateau; suggests constraint/feedback control	Confusing plateau with failure; not separating yeast limitation from ecological inhibition
Spatial microenvironments (architecture → modulation)	Oxygen/moisture gradients; habitat heterogeneity	Imaging, micro-sensors; structured sampling (surface vs. core)	Gradient proxy (surface–core difference in O₂/moisture/pH)	Explains niche partitioning and stability; links matrix to coupling regime	Treating solid systems as well-mixed; no spatial sampling
Causal validation (translation)	Necessary vs. sufficient architectural features	Reconstruction of minimal consortia; controlled re-inoculation	“Remove/restore” tests on trajectory metrics	Moves beyond correlation; identifies design rules for starter control	Over-simplification that breaks topology; ignoring matrix dependence

LAB, lactic acid bacteria; EPI, ethanol plateau index; LC, liquid chromatography; qPCR, quantitative PCR.

### 5.5 Safety constraints and toxigenic trajectories

These safety constraints represent an often-overlooked counterpart to the ecological flexibility of *Xiaoqu* (Dai et al., 2024). While open and functionally redundant microbial architectures may contribute to fermentation robustness and sensory complexity, they also introduce safety constraints associated with uncontrolled fungal succession and potential mycotoxigenic trajectories (Xu Q. et al., 2025). In *Xiaoqu* systems, filamentous fungi are essential drivers of saccharification, matrix restructuring, and ecological assembly, yet some environmental or contaminating molds may possess toxigenic potential under favorable conditions. Importantly, toxigenic risk should not be interpreted solely as the presence or absence of particular taxa, because secondary-metabolite production is strongly shaped by community interactions, substrate composition, moisture, oxygen availability, temperature, and stress-related metabolic states. Thus, toxin emergence represents not only a compositional issue but also a trajectory-level ecological phenomenon.

From an architecture-to-trajectory perspective, toxigenic states may emerge when normal assembly constraints and feedback controls become destabilized. Disrupted succession, excessive drying stress, abnormal oxygen penetration, nutrient imbalance, or weakened competitive exclusion may permit opportunistic fungi to proliferate or activate secondary-metabolite pathways. Conversely, stable community assembly and inhibitory interaction regimes may suppress toxin expression even in taxonomically diverse systems (Zhang H. et al., 2022; Zong et al., 2026). This distinction is important because it suggests that safety cannot be inferred solely from taxonomic inventories; rather, safety depends on whether the fermentation trajectory maintains ecological constraints that prevent activation of undesirable metabolic states.

These considerations also reveal an important trade-off inherent to open mixed-culture fermentations. The same ecological flexibility that supports robustness, flavor diversity, and adaptation to variable raw materials may simultaneously increase vulnerability to unstable or stress-induced trajectories. Therefore, modernization strategies for *Xiaoqu* should not aim only to preserve functional coupling and sensory complexity, but also to establish safety-oriented assembly filters that constrain toxigenic emergence while maintaining desirable metabolic modulation.

Operationally, architecture-preserving control may incorporate both process and ecological safeguards, including controlled moisture and drying regimes, environmental management during starter maturation and storage, exclusion of undesirable fungi through competitive community stabilization, and trajectory-oriented monitoring of fungal succession and metabolite accumulation (Jiang et al., 2026). Future studies integrating strain-level metagenomics, transcriptomics, metabolomics, and toxin-targeted analytics will be essential for distinguishing harmless fungal diversity from ecologically activated toxigenic states and for identifying which assembly conditions reproducibly suppress toxin-associated trajectories in *Xiaoqu*-based fermentations (Xu Q. et al., 2025).

## 6 Conclusion

*Xiaoqu*-based fermentation illustrates a central principle that is often obscured by species-centric narratives: robust glutinous rice wine production can be achieved through community-level organization rather than strict inoculum definition. When viewed as a process-shaped microbial architecture, *Xiaoqu* integrates saccharification, fermentation, and acidification into a coupled system in which molds, yeasts, and bacteria jointly construct—and respond to—dynamic microenvironments. This coupling offers a mechanistic basis for characteristic glutinous rice wine phenotypes, including rapid sugar release with sweetness retention, moderated acidification, and restrained ethanol accumulation, even under variable raw materials and ambient conditions. The key implication is that performance resides less in static membership than in guild partitioning, interaction topology, and functional redundancy, which together stabilize fermentation trajectories. This explains why taxonomic inventories alone rarely predict outcomes, and why single-strain substitutions frequently reproduce individual functions yet fail to reproduce the coupled metabolic trajectories that define *Xiaoqu*-mediated fermentations.

Translating this ecological logic into actionable design principles requires a decisive shift from correlation to causality. Progress will depend on integrative approaches that anchor multi-omics to time-resolved fermentation kinetics and metabolite trajectories, quantify guild structure and redundancy, and explicitly capture spatial organization and microenvironmental gradients within starters and fermentations. A particular priority is to move beyond “who is there” toward “what is doing what, when, and where,” linking enzyme activities, growth dynamics, and interaction regimes to sugar–acid–ethanol coupling and to trajectory-level endpoints relevant to sweetness retention and sensory balance. Experimentally reconstructing minimal yet functional consortia—guided by guild roles and interaction constraints rather than species lists—will be essential for identifying architectural features that are necessary and sufficient to reproduce desired metabolic modulation in glutinous rice wine systems, and for revealing which aspects of *Xiaoqu* architecture are robust to substitution versus those that are fragile to simplification.

These insights point to a pragmatic modernization pathway centered on architecture-preserving control. Rather than pursuing strict taxonomic standardization, controllable starter strategies should aim to preserve the ecological advantages of *Xiaoqu*, including resilience, sensory richness, and tolerance to process variability, while improving reproducibility, safety, and suppression of undesirable toxigenic trajectories. Operationally, this implies shifting quality targets toward architecture- and trajectory-relevant indicators, including saccharification timing, pH kinetics, ethanol plateau behavior, and ecological conditions associated with stable fungal succession and suppression of toxin-related metabolic states. It also supports the development of modular starter strategies—whether traditional, hybrid, or minimal consortia—provided that they preserve interaction topology, maintain safety-oriented assembly constraints, and reproduce the desired sugar–acid–ethanol coupling regimes. More broadly, treating *Xiaoqu* as an emergent microbial system shaped by processing and environment provides a transferable conceptual template for designing robust and safe solid-state fermentations beyond glutinous rice wine, including gluten-free and plant-based products where microbial diversity can be leveraged as a route to stable function under weak control.
